# Recovery of Co(ii), Ni(ii) and Zn(ii) using magnetic nanoparticles (MNPs) at circumneutral pH†

**DOI:** 10.1039/d4en01176g

**Published:** 2025-02-19

**Authors:** Katie E. B. O'Neill, Jagannath Biswakarma, Rich Crane, James M. Byrne

**Affiliations:** a School of Earth Sciences, University of Bristol Bristol BS8 1TH UK james.byrne@bristol.ac.uk; b Camborne School of Mines, University of Exeter Penryn TR10 9EZ UK

## Abstract

Growing demand for metals, particularly those with irreplaceable utility within renewable energy technology dictates an urgent demand for the development of new innovative approaches for their extraction from primary and secondary sources. In this study, magnetic nanoparticles (MNP) were investigated for their ability to remove cobalt (Co^2+^), nickel (Ni^2+^), and zinc (Zn^2+^) ions from neutral pH aqueous solutions under anoxic conditions. A MNP suspension (1 g L^−1^ or 5 g L^−1^) was exposed to varying concentrations of Co(ii), Ni(ii), and Zn(ii) (10–1000 mg L^−1^) in both single and mixed systems for 48 hours at pH 7.0 ± 0.1. Results show that MNPs can remove these ions to low concentrations (*K*_d_ values: Zn: 0.07 L g^−1^; Co: 0.02 L g^−1^; and Ni: 0.01 L g^−1^ in single metal systems). Transmission Electron Microscopy (TEM) analysis confirmed relatively homogenous surface coverage of MNPs by each metal, while X-ray Absorption Spectroscopy (XAS) measurements determined sorption *via* the formation of coordinate bonds between the sorbed metals and surface oxygen atoms (Fe–O). Overall, our results show that MNPs can serve as an effective and reusable sorbent for Zn, Ni and Co ions from circumneutral pH waters.

## Abstract 2

Growing demand for metals, particularly those with irreplaceable utility within renewable energy technologies dictates an urgent demand for the development of new innovative approaches for their extraction from primary and secondary sources.

## Graphical abstract

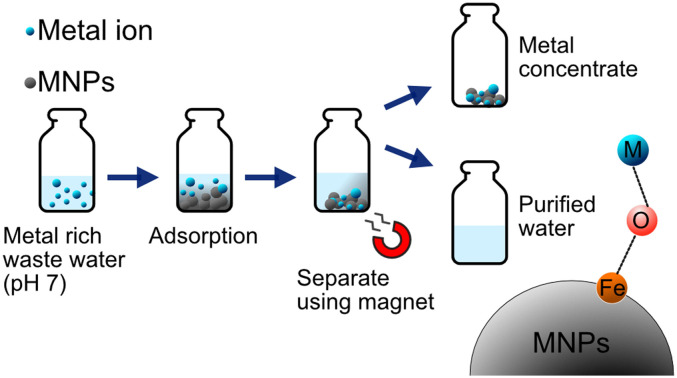

**Environmental significance**

With growing demand for metals in industries like renewable energy, electronics, and catalysis, sustainable methods for extracting metals from secondary waste sources are crucial. Understanding the sorption mechanisms of economically important Co, Ni, and Zn onto MNPs—such as electron transfer, ion exchange, and electrostatic interactions—can optimize the recovery processes of these elements, enhance selectivity for target metals, and promote sustainable recovery technologies while reducing reliance on primary resources and improving water quality.

## 1. Introduction

Global demand for critical and strategically important metals is escalating, in part due to their essential role in renewable and clean energy technologies. Consequently, resource scarcity and environmental concerns associated with conventional extraction methods have intensified.^1–4^ At the same time, significant metal losses into waste streams further exacerbate the challenge, highlighting the urgent need for the development of new metal recovery technologies.^5–10^

Co, Ni, and Zn are integral to various industrial processes, including electroplating as well as the production of alloys, magnets and batteries. As a result, demand for Ni, Co and Zn is set to rise between 60–80% (by 2050).^11,12^ As a consequence, these metals are commonly found in various types of wastewater, including mining effluents and industrial leachates. For example, 7.42–7.63 mg L^−1^ of Ni and 37.93–43.91 mg L^−1^ of Zn have been recorded in the Kor River, Iran, which is fed by numerous anthropogenic sources (domestic and industrial wastewater).^13^ Co, Ni and Zn are also prevalent in acid mine drainage (AMD), which is produced when sulfide-bearing minerals are exposed to air and water, and therefore a widespread environmental issue.^14^ In the UK, for example, despite the large-scale cessation of mining several decades ago, AMD continues to significantly impact water quality. Hundreds of kilometers of streams and rivers (totaling 6% of all surface waters) are impacted by discharges from abandoned metal mines, contributing hundreds of tons of soluble metals annually into receiving water bodies.^15^ Globally, several studies point to elevated concentrations of Co in rivers, with one Cu-Co plant in Zambia releasing discharges containing 34 400 mg Co L^−1^.^16^ In Uganda, the River Nyamwamba and its tributaries from the Kilembe mine contain elevated Ni and Co concentrations of 0.23 mg L^−1^ and 1 mg L^−1^ respectively.^17^

While AMD is inherently acidic, much of this water becomes pH neutralised in receiving waters, leading to the precipitation of poorly soluble metals, such as iron (Fe). However, certain metals, including Co, Ni and Zn are known to often remain at moderately elevated concentrations in such conditions,^18^ presenting an environmental liability but also a long-term metal resource opportunity.^19–23^

Circumneutral pH mine water can also be derived from non-acidic origins, such as in the absence of pyrite and due to the oxidation of metal sulphides (*e.g.* sphalerite or galena) that do not result in acidity. This type of mine water is highly prevalent world-wide, of which Zn is a common constituent. For example, in the UK alone there is an estimated 170 tons of Zn per year reported within such water.^24^ Given their often comparatively lower environmental impact compared to AMD, circumneutral pH mine waters are relatively understudied, and technology for metal recovery from such water remains relatively unexplored.^19–23^

Research to date on the recovery of Co, Ni and Zn from circumneutral pH waters has included membrane filtration, ion exchange, precipitation and adsorption.^25,26^ Within this, adsorption has emerged as a highly promising approach due to its high efficiency, versatility, low-cost, simple application and potential ability to be regenerated/reused. Adsorbents tested have including fly ash, wood ash and thermally active dolomite, however, test conditions are often limited to simplified systems containing only one target metal.^27–29^

Alternatively, magnetic nanoparticles (MNPs) show great potential for both individual and mixed metal systems, with favourable attributes including a large surface area to volume ratio, multiple active sorption sites, and (superpara)magnetic properties to enable efficient separation from the aqueous phase. MNPs are also naturally occurring and can form *via* both biogenic and geogenic processes,^26,30–32^ thus affording the possibility for natural routes towards MNP synthesis and/or the development of potentially environmentally compatible applications. Despite such promise, research within this field remains largely unexplored. Key publications include those where MNPs have been determined as effective in removing contaminants such as phosphorus (P), copper (Cu), arsenic (As) and cadmium (Cd) from water.^33–38^ For instance, Li *et al.*, reported 92.3% P removal using MgO-coated Fe_3_O_4_@SiO_2_ nanoparticles.^39^ While Bayer *et al.*, found that microbially reduced MNPs achieved adsorption capacities of 632 μmol g^−1^ Fe for Cd and 530 μmol g^−1^ Fe for Cu at pH 7.3.^40^ These findings highlight the versatility of MNPs for contaminant removal.

Such previous studies have predominantly focused on examining the application of MNPs for metal removal within single or binary metal systems.^30,31,33–35^ Therefore a key knowledge gap remains regarding the competitive sorption behavior of MNPs in multi-metal systems at circumneutral pH.

This study has been designed to determine the efficacy of MNPs as an adsorbent for removal of Co(ii), Ni(ii) and Zn(ii) at circumneutral pH using batch experiments under anoxic conditions. Specific objectives were to (i) identify the maximum concentrations which Co(ii), Ni(ii) and Zn(ii) ions could be removed in single and mixed metal systems onto MNPs, (ii) determine the extent of any differential sorption of Co(ii), Ni(ii) and Zn(ii) onto MNPs, (iii) elucidate the sorption mechanisms of Co(ii), Ni(ii) and Zn(ii) onto MNPs. The overarching aim of this study is therefore, to improve our fundamental mechanistic understanding of the interactions between Co(ii), Ni(ii) and Zn(ii) ions with MNPs in different systems to thereby further efforts to remediate and extract such strategically important metals from circumneutral pH waters.

## 2. Methods

### 2.1. MNP synthesis

MNPs were synthesized using a modified protocol outlined by Pearce *et al.* 2012 under ambient conditions.^41^ In a fume hood whilst being flushed with N_2_, FeCl_2_·4H_2_O (0.1 M) and FeCl_2_·6H_2_O (0.2 M) in HCl (0.3 M) were added dropwise into a solution of NH_4_OH (20%) being stirred at ∼800 rpm. A black precipitate immediately formed and after all contents were added the solution containing the black precipitate was sealed and degassed with N_2_ (100%) for 20 minutes to ensure it was anoxic (whilst continuing to be stirred). The anoxic MNP suspension was loaded into an anoxic chamber (COY, 98% N_2_, 2.1% H_2_) and then washed twice using ultrapure deionized water (Purite, 18 MOhm). Samples of the MNPs were taken for characterization by ferrozine assay, X-ray Diffraction (XRD), ^57^Fe Mössbauer spectroscopy, Transmission Electron Microscopy (TEM) and Brunauer Emmett Teller (BET) surface area analysis.

### 2.2. MNP characterisation

The total iron (Fe(Tot)) and ferrous iron (Fe(ii)) concentrations of the MNPs were measured in triplicate using the spectrophotometric ferrozine assay method.^42^ Samples were dissolved in 6 M HCl for 24 hours at room temperature inside an anoxic chamber. This was followed by a series of dilutions using 1 M HCl to within the calibration range of the iron standard ((NH_4_)_2_Fe(SO_4_)_2_·6H_2_O). Fe(iii) was calculated by subtracting Fe(ii) from Fe(Tot) concentrations. Spectrophotometric measurements were collected using a UV/VIS Multiskan SkyHigh Microplate spectrophotometer, wavelength 562 nm, and processed using SkanIt software.

XRD measurements were performed using a Bruker D8 Advance with a PSD LynxEye detector and Cu radiation (1.5406 Angstrom). Samples were put onto low background silicon wafer holders and the data was collected using the following parameters: angle range 5–75°, step size 0.02° and 1 second dwell time. Samples were compared against a database to confirm the mineral phase.

Particle size and morphology were determined by Transmission Electron Microscopy (TEM). MNPs were prepared by centrifuging 1 mL from a stock in an anoxic chamber. For analysis of the sorption experiments, 1 mL of sample was collected from the 1000 ppm initial concentration reactors, (for both individual and mixed metals). These were centrifuged and the supernatant was discarded and 1 mL of 20 mM NaCl added. 200 μL of each sample was then placed onto a copper TEM grid coated with approximately 5 nm of graphite and left to dry anoxically. Samples were imaged on a field- emission gun JEM-2100F from JEOL at 200 kV. EDX data was collected in STEM mode using an X-Max 80 mm^2^ EDX detector and analysed in Aztec software both from Oxford instruments.

BET method was used to determine the specific surface area (SSA). In an anoxic chamber MNPs were centrifuged and the supernatant was removed. The material was dried in an oven at 80 °C for 4 hours and 120 mg of dried MNPs was collected for analysis. This was then left in the chamber to dry. The SSA was measured using an Anton Paar autosorb iQ gas sorption analyser with N_2_ as the adsorbate.

^57^Fe Mössbauer spectroscopy was conducted on a sample of the synthesised MNPs. Under anoxic conditions 1 mL of sample from a stock was centrifuged and the supernatant discarded. The pellet was dried in the anoxic chamber (COY, 98% N_2_, 2.1% H_2_) for 6 hours. After this time, the sample was put onto a filter paper and sealed between two layers of Kapton tape. The sample was transferred into a closed-cycle exchange gas cryostat (Janis cryogenics) under a backflow of helium. The measurement was made at 295 K with a constant acceleration drive system (WissEL) in transmission mode with a 57Co/Rh source and calibrated against a 7 μm thick α-57Fe foil measured at room temperature. All spectra were analyzed using recoil (University of Ottawa) by applying a Voight Based Fitting (VBF) site analysis. The half width at half maximum (HWHM) was fixed to a value of 0.15 mm s^−1^.

### 2.3. Sorption experiments

Adsorption experiments were carried out to investigate the interaction between MNPs and the divalent metals. Solutions were prepared using chemical reagents of analytical grade: CoCl_2_·6H_2_O, NiCl_2_·6H_2_O, and ZnCl_2_. All experiments were performed in triplicate. Four stock solutions of 30 000, 10 000, 4000 and 1000 mg L^−1^ were prepared for each metal individually and additional concentrations were obtained by dilution using ultrapure deionised water, HEPES buffer (10 mM) and NaCl (10 mM) as an electrolyte in each reactor bottle. The sorption experiments were performed with a total volume of 20 mL at ambient temperature and pH 7. The concentrations of MNPs used were 1 g L^−1^ and 5 g L^−1^ with a contact time of 48 hours. In a single metal system, initial concentrations (*C*_i_) of metal were varied at 10, 50, 100, 250, 500 and 1000 mg L^−1^. Similarly, in a mixed metal system experiments were performed under the same conditions but with different varying initial concentrations of metal 20, 40, 100, 200, 400 and 800 mg L^−1^. Samples were collected at the start and end of the 48 hours by centrifugation at 5000 rpm for 5 minutes. The supernatants were collected and the solid fraction was stored under anoxic conditions. The metal concentration of each supernatant (*C*_e_) was determined by an Agilent 5110 ICP-OES with the following operating conditions: a plasma argon flow rate at 12 L min^−1^, auxiliary flow rate at 1 L min^−1^, read time at 5 seconds, nebulizer argon flow rate at 0.7 L min^−1^. The viewing mode was axial. A calibration curve for each element was established from the respected standard stock solutions. The linearity for each element had good *R*^2^ values ∼0.99.

Data collected after sorption experiments were plotted with Log transformed values. A linear regression line (equivalent to the Log linearised form of the Freundlich equation) was used to fit the data.1
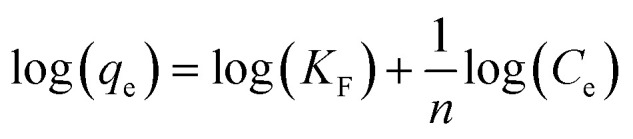
where:

*q*_e_ is the amount of adsorbate adsorbed per unit mass of adsorbent (mg g^−1^),

*C*_e_ is the equilibrium concentration of the adsorbate in the solution (mg L^−1^),

*K*_F_ is the Freundlich adsorption coefficient indicating the capacity of the adsorbent,

1/*n* is a dimensionless constant related to the intensity of the adsorption process.

From this equation we extracted sorption parameters *K*_F_ and 1/*n*. Since the data followed a linear trend, we also plotted partition coefficients (measured *K*_d_ = *q*_e_/*C*_e_). We also calculated the *K*_d_ value based on the sorption parameters obtained from eqn (1) using:

2 log(*K*_d_) = log(*q*_e_) − log(*C*_e_)

3
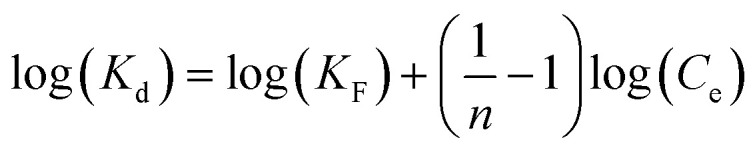


### 2.4. Co(ii) or Ni(ii) sorbed on MNPs through X-ray absorption spectroscopy

XAS analysis was conducted at beamline BM28 (XMaS), of the European Synchrotron Radiation Facility (ESRF) in Grenoble, France. Samples were made from the solid fraction collected after each sorption experiment only for single metal systems. Wet pastes for each sample were sealed between Kapton tape inside the glovebox and mounted into the XAS sample holder. X-ray absorption near-edge structure (XANES) spectra were recorded at Fe (7112 eV), Co (7709 eV), and Ni (8333 eV) K-edges in an N_2_ environment. Zn samples were not measured with XAS. We successfully distinguished the emission lines of Fe K_α1_, K_α2_, K_β_, and Co K_α_ for collecting the Fe (XANES; transmission mode) and Co (XANES, fluorescence mode) data. XANES spectra and pre-edge spectra were recorded with 0.1 eV step. Data were collected using Si-drift diodes (Ketek) detector. Multiple scans (4–8) were recorded for each sample depending on the data quality and to improve the signal-to-noise ratio. All scans of each sample were carefully checked for any beam-induced changes, which were not observed. Data were processed and analysed with Athena 0.9.26. Spectra of each measured sample were merged, energy shift corrected, and *E*_0_ set by selecting the maximum intensity of the first derivative. Merged spectra were normalized using the Autoback routine of Athena (0.9.26).^43^

## 3. Results and discussion

### 3.1. Characterisation

**Fig. 1:**
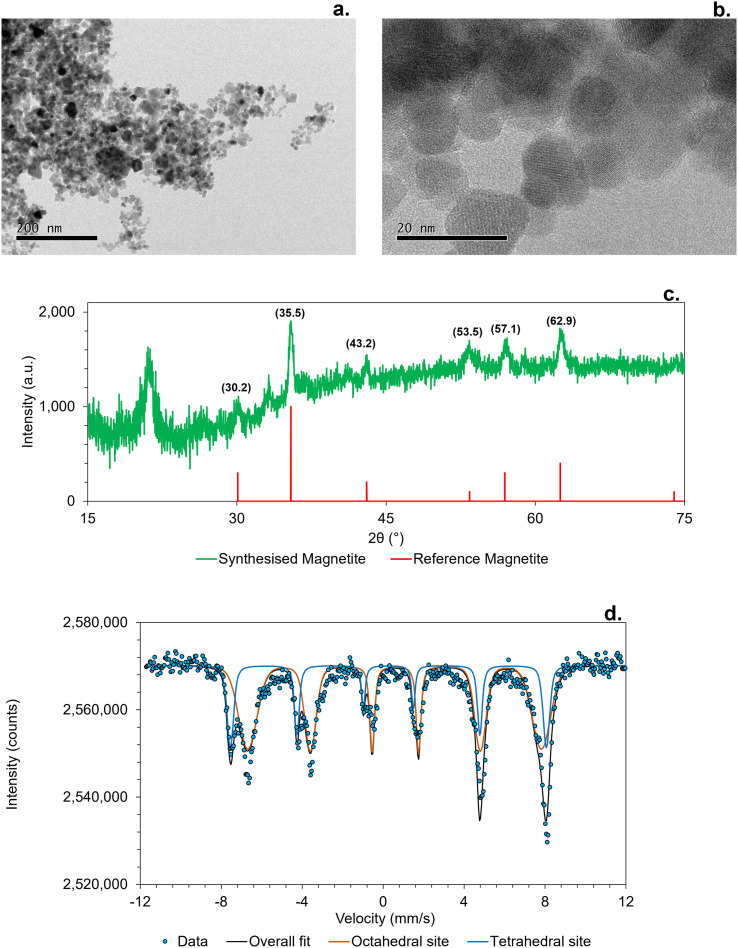
Characterization of synthesised MNPs. a) Transmission Electron Microscopy (TEM) of MNPs at 200 nm scale and b) at 20 nm scale, c) depicts X-Ray Diffraction (XRD) reflections of MNPs with the synthesised sample in green and a reference magnetite reference in red. XRD measurements were obtained using the Cu radiation (*λ* = 1.5406 Å). d) Mössbauer spectra of synthesised MNPs at room temperature (295 K).

Synthesized MNPs were characterized by several techniques. As shown in Fig. 1a and b, TEM micrographs show the synthesised MNPs at two magnifications (*a* = 200 nm, *b* = 20 nm). Low magnification imaging (Fig. 1a) revealed aggregated clusters of MNPs, whereas high magnification imaging (Fig. 1b) showed spherical particles estimated to have an average size of 15 nm ± 6 (*N* = 159 particles) as calculated using Image J. This morphology and nanoscale suggests a high surface area that was confirmed by BET analysis which recorded the specific surface area as 70 m^2^ g^−1^.

Fig. 1c displays the XRD pattern of the MNPs in green compared against a reference magnetite in red. The diffraction reflections at 30.2°, 35.5°, 43.2°, 53.5°, 57.1° and 62.9° corresponded to that of magnetite ((220), (311), (400), (422), (511) and (440) crystal planes^44,45^), confirming the purity of the material. This is also in agreement with M*ö*ssbauer spectroscopy (Fig. 1d) which displays two distinct sextets corresponding to Fe in octahedral (orange) and tetrahedral (blue) coordination sites. The Fe(ii)/Fe(iii) ratio was calculated from the M*ö*ssbauer spectrum to be 0.52 (Table S1†) which closely aligns with the ratio of 0.49 ± 0.007 determined by Ferrozine assay. These results demonstrate the successful synthesis of MNPs with high crystallinity, uniform particle size distribution and an Fe(ii)/Fe(iii) ratio consistent with magnetite.^45^

### 3.2. Metal sorption onto MNPs in single and mixed systems

**Fig. 2:**
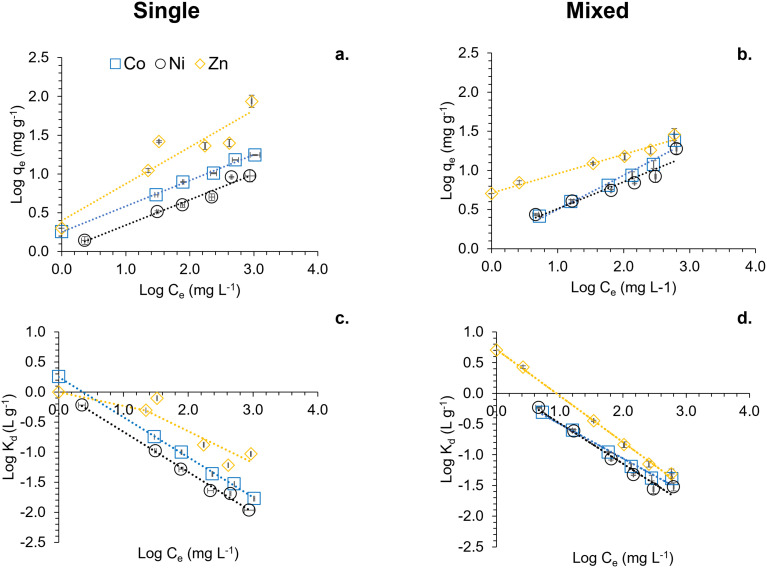
Sorption of Co(ii), Ni(ii) and Zn(ii) on MNPs (5 g L^−1^) at pH 7 under anoxic conditions. Log transformed data is presented. Logarithmic forms of the data collected for sorbed fraction (log *q*_e_) and dissolved equilibrium concentration (log *C*_e_) are shown in Fig. 2a and b. 2a shows single metal experiments while 2b a mixed metal system. Dotted lines represent the linearised form of log transformed model data calculated using the Freundlich equation, as shown in eqn (1) in the main text. 2c and d demonstrate the log-transformed distribution coefficient (log *K*_d_) for single and mixed metal systems respectively. Co(ii) is shown as blue squares, Ni(ii) as black circles and Zn(ii) as yellow diamonds. Dotted lines represent the modelled log *K*_d_ values obtained from eqn (3). Error bars represent standard deviations collected from triplicate experiments.

Sorption experiments were conducted to elucidate the interaction between MNPs (suspension concentration 1 and 5 g L^−1^) and Co(ii), Ni(ii), and Zn(ii) at pH 7.0 under anoxic conditions. These experiments included both single metal systems, with metal concentrations of 50, 100, 250, 500, and 1000 mg L^−1^, and mixed metal systems, with concentrations of 20, 40, 100, 200, 400, and 800 mg L^−1^ for each metal. Fig. 2 illustrates the sorption behaviour of Co(ii), Ni(ii), and Zn(ii) on MNPs (5 g L^−1^; see Fig. S1† for 1 g L^−1^ data) at pH 7.0 under anoxic conditions, with Log-transformed data for both single and mixed metal systems.

Fig. 2a and b present the logarithmic values of the sorbed fraction (log *q*_e_, mg g^−1^) against the logarithmic values of the dissolved equilibrium concentration (log *C*_e_, mg L^−1^). Within the applied experimental conditions, as the dissolved equilibrium concentration increases, the sorbed fraction linearly increases, highlighting the sorption capacity of each metal on MNPs. In the single metal experiments (Fig. 2a), each metal's sorption is individually assessed, showing distinct linear trends represented by the dotted lines corresponding to the linearized Log-transformed Freundlich equation (eqn (1)). The steeper slopes for Zn(ii) indicate a higher sorption capacity compared to Co(ii) and Ni(ii). In contrast, Fig. 2b shows the sorption in a mixed metal system, highlighting competitive interactions among the metals. The linear fits show deviations, particularly for Co(ii) and Ni(ii), suggesting that the presence of multiple metals affects their sorption dynamics on MNPs. The linearized equations benefit us in extracting sorption parameters such as *K*_F_ and *n*, as listed in Table 1. These parameters were employed to determine the distribution coefficients, as described in the Material and Methods Section.

**Table 1:** Sorption experiments: distribution coefficient (*K*_d_ in L g^−1^) calculated for metal concentrations *C*_e_ of 1000 mg L^−1^

Mineral	Metal	Single/mixed	Mineral concentration (g L^−1^)	pH	*K*_d_ (L g^−1^) *C*_e_ = 1000 mg L^−1^^a^	Reference
Magnetite	Co	Single	5	7	0.02	This study
Magnetite	Ni	Single	5	7	0.01	This study
Magnetite	Zn	Single	5	7	0.07	This study
Magnetite	Co	Mixed	5	7	0.02	This study
Magnetite	Ni	Mixed	5	7	0.02	This study
Magnetite	Zn	Mixed	5	7	0.03	This study
Magnetite	Zn	Single	4	6	0.05	46
Magnetite–baobab composite	Zn	Single	4	6	0.04	46
Baobab	Zn	Single	4	6	0.02	46
Magnetite graphene oxide composite	Co	Single	0.4	6.8	0.01	47
Spirulina	Co	Single	1	6	0.10	48
Activated charcoal	Co	Single	1	6	0.05	48
Chitosan-magnetite nanocomposite	Ni	Single	2	6	0.03	49
Chitosan-magnetite nanocomposite	Co	Single	2	6	0.05	49
2,4-Dinitrophenylhydrazine (DNPH)	Co	Single	1	5	0.04	50
2,4-Dinitrophenylhydrazine (DNPH)	Ni	Single	1	5	0.02	50
Kaolinite (Prosyanov)	Ni	Single	Solid : Liquid ratio 1 : 100	7	0.06	51
Meta-kaolinite	Ni	Single	Solid : Liquid ratio 1 : 100	7	0.07	51
Aluminium oxyhydroxide, peptised	Ni	Single	Solid : Liquid ratio 1 : 100	7	0.10	51
Aluminium hydroxide (Al_13_-gel)	Ni	Single	Solid : Liquid ratio 1 : 100	7	0.18	51

a *K*_d_ values are calculated from eqn (3) when Freundlich parameters *K*_F_ and 1/*n* were available. Conversely, when *Q*_max_ was found, *K*_d_ were determined by assuming *Q*_max_ ≈ *q*_e_ and using equation *K*_d_ = *q*_e_/*C*_e_ (*C*_e_ = 1000 mg L^−1^).

Fig. 2c and d illustrate the distribution coefficients (log *K*_d_, L g^−1^) for the single and mixed metal systems, respectively. The experimentally obtained log *K*_d_ using eqn (2) was plotted against log *C*_e_. The dotted lines in Fig. 2c and d represent the calculated log *K*_d_ values from eqn (3), showing that both measured and calculated values are complementary. This indicates that the distribution coefficient (*K*_d_) is a reliable descriptor of the sorption behaviour of the collected dataset. In Fig. 2c, the log *K*_d_ values for single metal systems highlight the relative affinity of each metal for MNPs, with Zn(ii) displaying the highest distribution coefficient, followed by Ni(ii) and Co(ii); also see Table 1.

Fig. 2d shows the log *K*_d_ values in the presence of all three metals, indicating higher preferential sorption for Zn(ii) at lower concentrations compared to Co(ii) and Ni(ii) whereas these two metals behave similarly in the same concentration range. The log *K*_d_ values of Zn(ii) decrease as the concentration increases whereas Ni(ii) and Co(ii) remain similar up to around 1.4 mg L^−1^ whereby the log *K*_d_ values of Co(ii) are less than those of Ni(ii). Overall, Zn(ii) shows marginally higher log *K*_d_ values than both Co(ii) and Ni(ii) in the mixed metal system and the single metal system. This suggests that Zn(ii) has a minor competitive advantage in binding to MNPs under both single and mixed metal conditions. The error bars, representing standard deviations from triplicate experiments, underscore the reliability of these observations.

Fig. 2 collectively demonstrates the sorption characteristics and distribution patterns of Co(ii), Ni(ii), and Zn(ii) on MNPs under varying conditions. The kinetics of sorption were found to be too rapid to capture within our experimental conditions (Fig. S2†).

Table 1 presents the distribution coefficients (*K*_d_) for Co, Ni, and Zn sorption onto MNPs (this and previous studies)^46,52,53^ and various composites (previous studies)^46–50^ calculated at dissolved equilibrium metal concentrations (*C*_e_) of 1000 mg L^−1^. Notably, the *K*_d_ values are higher at the lower metal concentration, indicating a stronger adsorption affinity when the solution is less saturated with metal ions. For instance, MNPs exhibits a marked decrease in *K*_d_ at pH 7.0 for all metals as *C*_e_ increases, as shown in Fig. 2c, reflecting the limited adsorption sites at higher concentrations. Adsorbents, such as aluminium hydroxide (Al_13_-gel) show enhanced adsorption capacities at pH 7.0, particularly for Ni (*K*_d_ = 0.18 L g^−1^) at *C*_e_ = 1000 mg L^−1^.^51^ Similarly, natural adsorbents like spirulina and activated charcoal also demonstrate moderate adsorption capabilities.^48^ Furthermore, modified adsorbents, such as magnetite graphene oxide composite perform comparatively to the MNPs in this study at a significantly lower concentration suggesting surface chemical modifications can substantially improve adsorption performance.^47^ Overall, the data underscores the critical influence of pH, mineral type, and surface modifications on adsorption efficiency, with composites and modified materials generally outperforming their pure counterparts. This highlights the potential for tailored adsorbents in environmental remediation applications.

### 3.3. Elemental distribution

**Fig. 3:**
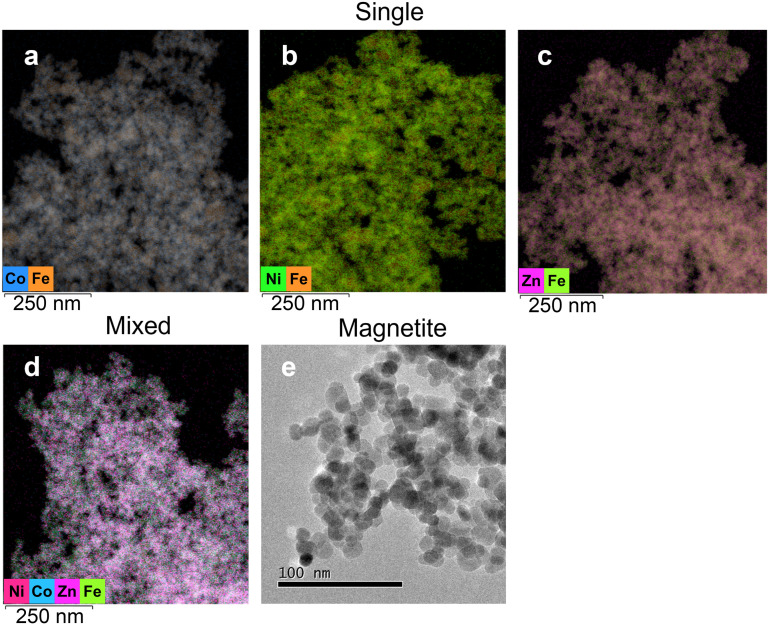
Transmission electron micrographs of solid samples collected after sorption of Co(ii), Ni(ii) and Zn(ii) on MNPs (5 g L^−1^) at pH 7 under anoxic conditions. Energy Dispersive X-ray spectroscopy (EDX) elemental mapping for single metal experiments show a) Co in blue b) Ni in green and c) Zn in pink covering the MNPs surface. In addition, Fig. 3d presents the mixed metal system where Co is blue, Ni is dark pink and Zn is light pink. Fig. 3e shows pure MNPs.

Electron microscopy coupled to elemental mapping (STEM-EDX) was performed to further understand the morphology and relationship between metals and the MNPs (Fig. 3). The results confirmed the association of Co, Ni and Zn with the nanoparticles, with a relatively even distribution of each metal on the surface of the MNPs (Fig. 3a–d). In the mixed metal system, Zn appeared to be the most dominantly visible metal covering the MNPs. However, Co and Ni can also be identified covering the surface as shown in the EDX map (Fig. 3d). Metal to Fe ratios were calculated based on EDX (Fig. S3†) to determine the amount of Fe required to remove metals from the solution. In the single metal system, the ratio of Co, Ni, and Zn to Fe were 0.044, 0.036, and 0.101. In contrast, in the mixed metal system, the ratios were found to be smaller, as Co, Ni, and Zn to Fe were 0.018, 0.012, and 0.042, respectively.

### 3.4. Speciation dynamics of sorbed Co(ii) and Ni(ii) with MNPs

**Fig. 4:**
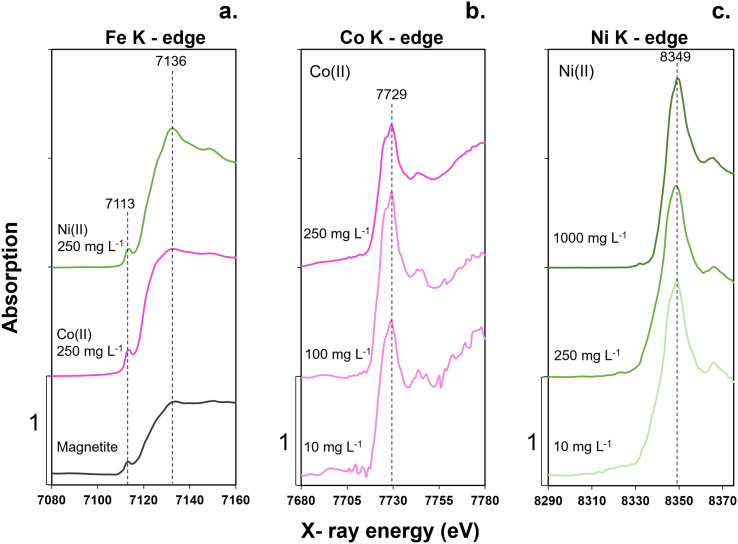
X-ray absorption near edge structure (XANES) spectra at the a) Fe K-edge, b) Co K-edge and c) Ni K-edge collected at 7112 eV, 7709 eV and 8333 eV, respectively. Varied concentrations of Co(ii) and Ni(ii) were treated with 5 g L^−1^ MNPs at pH 7 under anoxic conditions for 48 h. Fig. 4a shows reference spectrum of magnetite and two MNP samples exposed to 250 mg L^−1^ Co(ii) and Ni(ii) at Fe K-edge. Fig. 4b shows a varied range of Co(ii) sorbed onto MNPs Co K-edge. Fig. 4c presents a varied range of Ni(ii) sorbed onto MNPs at Ni K-edge.

We utilized X-ray absorption spectroscopy (XAS) to investigate changes in metal ion speciation and potential structural perturbations in MNPs upon sorption of Co and Ni. Fig. 4 shows processed XANES spectra obtained in the Fe, Co, and Ni energy regions.

Analysis of Fe spectra (Fig. 4a) indicated that the sorption of Co and Ni did not result in clear observable structural changes in the MNPs. However, the addition of 250 mg L^−1^ of Ni(ii) visibly altered the shape of the post-edge region (7137–7160 eV) of the MNPs, indicating the formation of Fe–O–Ni bonds. Conversely, the addition of 250 mg L^−1^ of Co(ii) did not lead to the visible changes in the spectrum compared to our reference magnetite sample, suggesting that the MNPs structural integrity was maintained even upon interaction with Co(ii). Future studies are required to understand the changes occurring in post edge region of these spectra. Nevertheless, the Fe K-edge XANES results demonstrate that the overall speciation of our MNPs with or without metal treatment remained consistent. Such stability is crucial in preserving the overall structure and functionality of the nanoparticles as a sorbent material. These observations highlight the robustness of MNPs as a sorbent, capable of hosting and interacting with metal ions without compromising its fundamental structural characteristics.

Fig. 4b and c show Co K-edge and Ni K-edge XANES spectra, respectively. These spectra demonstrate the sorption of Co and Ni onto MNPs at varying concentrations. The peak position in the spectra remained the same, indicating that there were no changes in the oxidation state within the applied experimental conditions. However, some changes were observed with respect to the references used and available literature, which requires further evaluation in a future study.

XANES spectra (Fig. 4b) indicated that Co formed bonds with surface-associated Fe species within the MNPs lattice. The Co speciation profiles exhibit a slight shift from 7724 eV, characteristic of Co(ii) ref. 54, to 7729 eV suggesting possible ternary complex formation with ligands available on the MNPs surface.

Similarly, Fig. 4c illustrates the XANES spectrum obtained within the Ni energy range. This spectrum demonstrates characteristic features consistent with Ni sorption onto MNPs across varying concentrations. The speciation of Ni exhibits subtle modifications, indicating that Ni also forms bonds with Fe species present on the MNPs surface without altering the mineral structure or undergoing speciation changes.

### 3.5. Mechanisms of Zn, Co and Ni ion removal by MNPs

Recovering divalent metals such as Co(ii), Ni(ii), and Zn(ii) through sorption on MNP surfaces at pH 7 involves a complex sequence of surface interactions governed by electrostatic forces and coordination chemistry. When in an aqueous solution, MNPs are negatively charged within the applied experimental condition due to the formation of outer sphere complexes, such as surface hydroxyl groups (≡Fe–OH). Co(ii), Ni(ii) and Zn(ii) are attracted to the negatively charged MNPs and can form inner sphere complexes whereby, metal ions interact with the hydroxyl groups on the MNPs, as explained below.

According to theoretical thermodynamic calculations using Visual MINTEQ 3.1, under neutral pH conditions, the fraction of metals that sorb increases with increasing concentrations of dissolved metals, up to 100 mg L^−1^ (Fig. S4†). Beyond this point, sorption saturation occurs, indicating that there are limited surface sites available for additional binding. Furthermore, pH dependence calculations highlight that for single metals, at a concentration of 500 mg L^−1^, the sorption of Co(ii) and Ni(ii) consistently increases with pH from 5 to 8 (Fig. S5a†). Conversely, Zn(ii) exhibits peak sorption within a narrower pH range of 6.5–7.2. In the mixed-metal system (Fig. S5b†), a broader pH range (5.8–12.5) facilitates maximum sorption for all tested metals, likely due to competitive interactions and differential adsorption site preferences. These findings are in good agreement with our experimental observations, as described in Fig. 2.

Furthermore, speciation analysis (Fig. S6†) reveals that the aqueous species such as CoOH^+^, NiOH^+^, CoCl^+^, and NiCl^+^ become increasingly prominent with higher applied concentrations of Co(ii) and Ni(ii) in solution. In the case of Zn(ii), a five fold higher concentration of aqueous species ZnCl^+^ compared to CoCl^+^, and NiCl^+^ was obtained and a two fold increase in ZnOH^+^ concentration compared to CoOH^+^ and NiOH^+^ could plausibly have led to higher sorption of Zn(ii) to the MNPs.

As the sorption process continues, Co(ii) and Ni(ii) undergo coordination with surface oxygen atoms (Fe–O) within the MNP lattice. This coordination involves the creation of stable metal–oxygen (M–O) bonds, leading to the formation of surface-bound species such as Fe–O–Co^+^ and Fe–O–Ni^+^ (Fig. S6 and S7†). These surface-bound complexes represent specific surface complexation modes and coordination chemistry, where metal ions are coordinated with the MNP structure through bonding interactions with available surface sites. This is supported by the XANES spectra (Fig. 4) showing an observed energy shift towards higher energy (to the right) upon sorption of Co(ii) and Ni(ii) on MNP surfaces. While sorption can influence the local chemical environment and coordination of Co or Ni, it generally does not involve significant speciation changes of these metals under ambient conditions.

In summary, Co(ii) and Ni(ii) sorption onto MNPs at pH 7 involves initial electrostatic attraction followed by surface complexation and coordination, forming stable surface-bound metal-oxygen complexes. The concentration-dependent sorption behavior and observed spectral shifts in XANES spectra provides the mechanisms of metal-MNP interactions, contributing to our understanding of environmental sorption processes and the potential applications of MNPs as (ad)sorbents for heavy metal contaminants.

Nevertheless, despite this study demonstrating the application of MNPs at pH 7.0 to extract Co(ii) and Ni(ii) under anoxic conditions, it is not representative of heterogeneous environmental conditions. As such, extrapolating these findings to real-world scenarios should be cautiously approached. Additionally, it should be considered that while Visual MINTEQ modeling offers predictions, it is based on specific assumptions that may not be appropriate for MNPs.

## 4. Conclusions

As demand for metals continues to increase there is an escalating need for the development of more efficient and sustainable methods to extract such metals from the aqueous phase. This includes the need to improve practices in primary ore mining and waste reprocessing, but also for environmental remediation. This study has documented MNPs as effective for the recovery of Co(ii), Ni(ii), and Zn(ii) ions from circumneutral pH waters. The findings demonstrate that MNPs effectively lower Zn, Co, and Ni concentrations to low levels, with distribution coefficients (*K*_d_) of 0.07 L g^−1^ for Zn, 0.02 L g^−1^ for Co, and 0.01 L g^−1^ for Ni in single-metal systems. TEM revealed a relatively uniform distribution of metals on the MNPs surface, while XANES identified that sorption occurs through the formation of coordinate bonds between the metals and surface oxygen atoms (Fe–O).

Further work is required to determine the commercial viability of this emerging water treatment technology, including economic and environmental impact evaluation. Regarding the former, in this study we applied co-precipitation to synthesize MNPs. This method was effective in producing high purity material with narrow size distribution and therefore could potentially be upscaled relatively easily and at low cost. Regarding the environmental implications of MNP use, their nanoscale properties and largely unknown environmental impact, necessitate caution to prevent their release into the natural environment.^55^ Although most applications of this technology are expected to be *ex situ*, there remains a risk of unintentional discharge into the environment. Within this, although substantial advancements have been made in the development of flow-through systems incorporating filtration, coagulation, and other retention mechanisms to minimize their presence in effluent wastewater,^56,57^ further research in this area is required.

## Data availability

Data supporting the findings of this study are available as a ESI† file.

## Author contributions

K. E. B. O.: conceptualization, methodology, investigation, writing – original draft, review & editing. J. B.: conceptualization, methodology, supervision, investigation, writing – original draft, review & editing, beamtime funding acquisition. R.C.: conceptualization, methodology, supervision, writing – review & editing, funding acquisition. J. M. B.: conceptualization, methodology, supervision, writing – review & editing, beamtime funding acquisition, project funding acquisition, project administration.

## Conflicts of interest

The authors declare no competing financial interest.

## Supplementary Material

EN-012-D4EN01176G-s001
