# Supplementary material for: Recovery of Co(ii), Ni(ii) and Zn(ii) using magnetic nanoparticles (MNPs) at circumneutral pH
Source: Environ Sci Nano. 2025 Feb 19;12(4):2371–82. doi: 10.1039/d4en01176g (PMC11863564; doi:10.1039/d4en01176g)
Supplement: EN-012-D4EN01176G-s001 [file EN-012-D4EN01176G-s001.pdf]

Supplementary Information (SI) for Environmental Science: Nano.
This journal is © The Royal Society of Chemistry 2025

Recovery of Co(II), Ni(II) and Zn(II) using magnetic nanoparticles at circumneutral pH

Katie E.B. O'Neill[1], Jagannath Biswakarma[1], Rich Crane[2], James M. Byrne[1*]

[1] School of Earth Sciences, University of Bristol, Bristol, BS8 1TH, United Kingdom

[2] Camborne School of Mines, University of Exeter, Penryn, TR10 9EZ, United Kingdom

[*] Corresponding author: james.byrne@bristol.ac.uk

Supporting Tables: 1

Supporting Figures: 7

Table 1 –Mössbauer spectroscopy results. δ – isomer shift; ε – quadrupole splitting; B_{hf} – hyperfine magnetic field; stdev(B_{hf}) – standard deviation of hyperfine magnetic field; R.A. – Relative abundance; Red. $\chi2$ – goodness of fit.

Site	δ (mm/s)	ε (mm/s)	B_{hf} (T)	stdev(B_{hf}) (T)	R.A. (%)	Error	Red. X2
Tetrahedral	0.269624	-0.00636	48.4443	0.356193	31.4	1.5	1.27
Octahedral	0.585783	-0.0138	45.0441	1.9387	68.6	1.5	

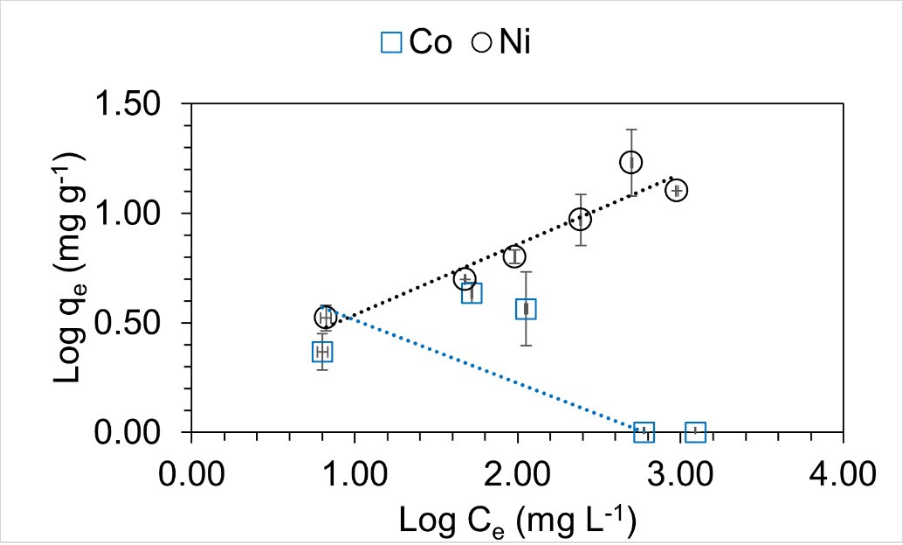

Figure S1: Sorption of Co(II) and Ni(II) on MNPs (1 g L^{-1}) at pH 7 under anoxic conditions. Log transformed data is presented. Logarithmic forms of the data collected for sorbed fraction (q_e, mg g^{-1}) and dissolved equilibrium concentration (C_e, mg L^{-1}) were shown in the Figure. Dotted lines represent the linearised form of the transformed Freundlich equation. Co(II) is shown as blue squares and Ni(II) as black circles. Error bars represent standard deviations collected from triplicate experiments.

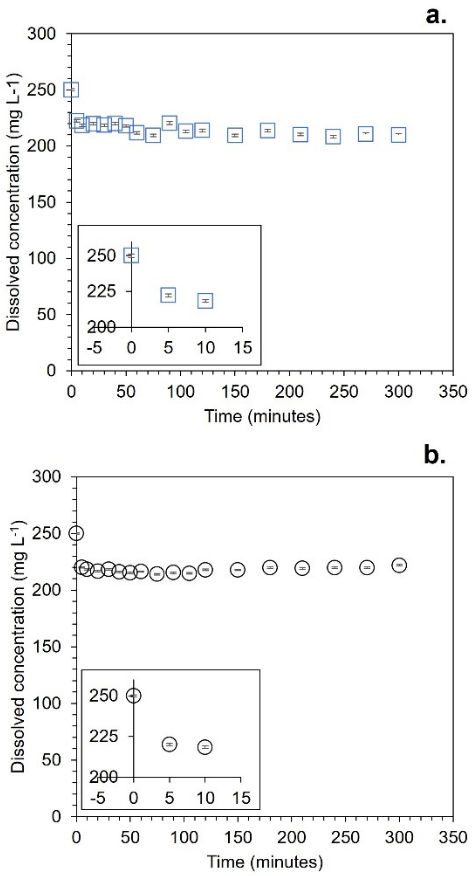

Figure S2: Kinetics of sorption of Co(II) and Ni(II) on MNPs (5 g L^{-1}) at pH 7 under anoxic conditions. Dissolved equilibrium concentration (mg L^{-1}) on the y- axis is plotted as a function of time (minutes) on the x-axis. Co(II) is shown as blue squares in 2a and Ni(II) as black circles in 2b. Error bars represent standard deviations collected from triplicate experiments.

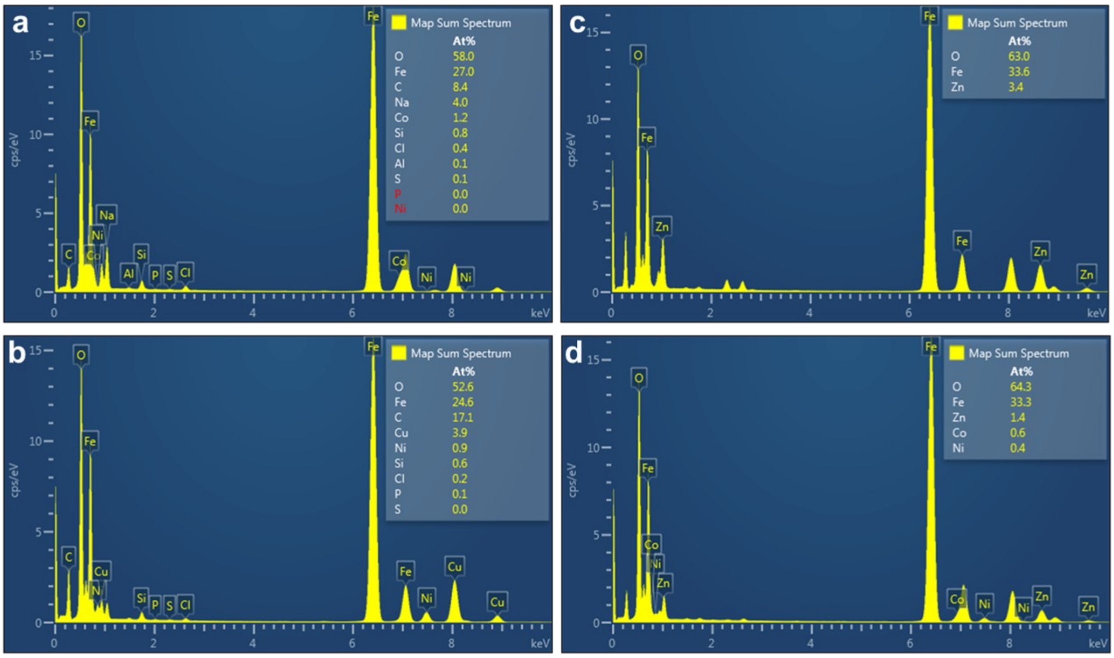

Figure S3: Electron Dispersive X-ray spectra (EDX) of solid samples collected after sorption of Co(II), Ni(II) and Zn(II) on MNPs (5 gL^{-1}) at pH 7.0 under anoxic conditions. Single metal experiments show a) Co b) Ni and c) Zn. In addition, Figure 3d presents the mixed metal system with Co, Ni and Zn.

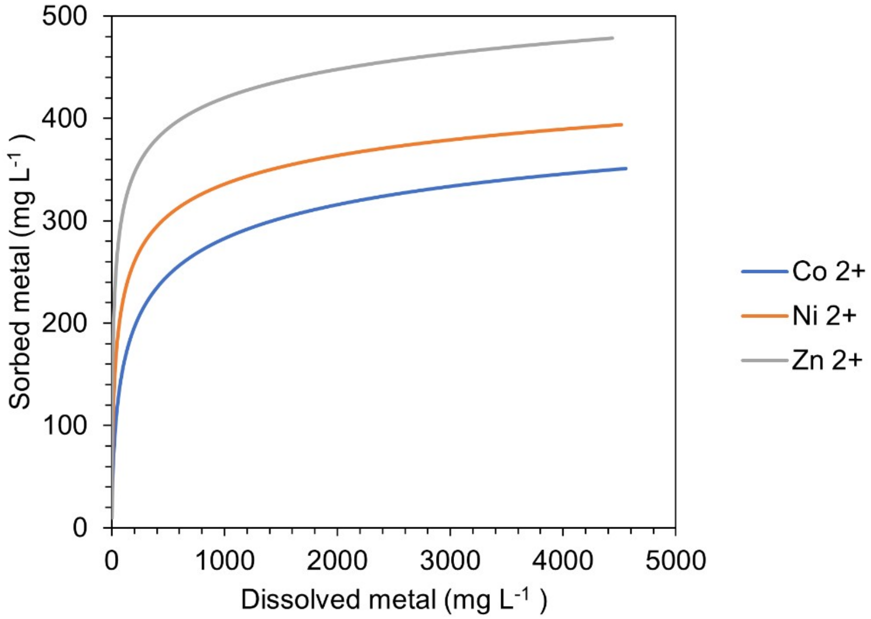

Figure S4: Theoretical sorption estimates by Visual MINTEQ. Co(II), Ni(II) and Zn(II) in separate experiments were exposed to iron via HFO model (5 g L^{-1}) at pH 7. Dissolved equilibrium concentration after sorption (y axis) and dissolved initial concentration in equilibrium (x axis). Co(II) is shown in blue, Ni(II) in orange and Zn(II) in grey.

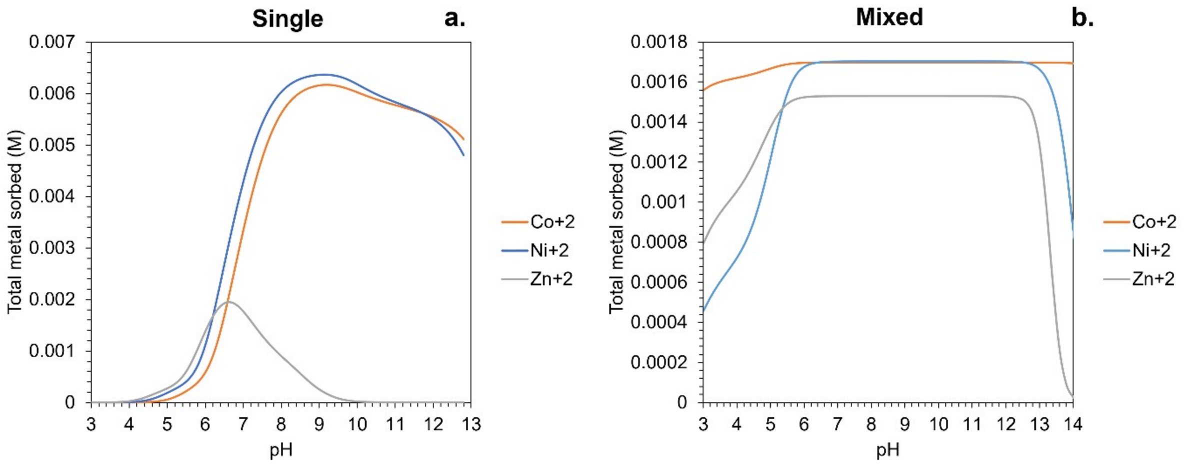

Figure S5: pH dependence sorption of Co(II), Ni(II) and Zn(II) in a) separate metal experiments and b) mixed metal experiments. The model was generated by visual MINTEQ 3.1 and used the HFO model (iron 5 gL^{-1}) at 500 mg L^{-1} of each metal. Total metal sorbed (y axis) as a function of pH. Co(II) is represented by the orange line, Ni(II) by the blue and Zn(II) by the grey.

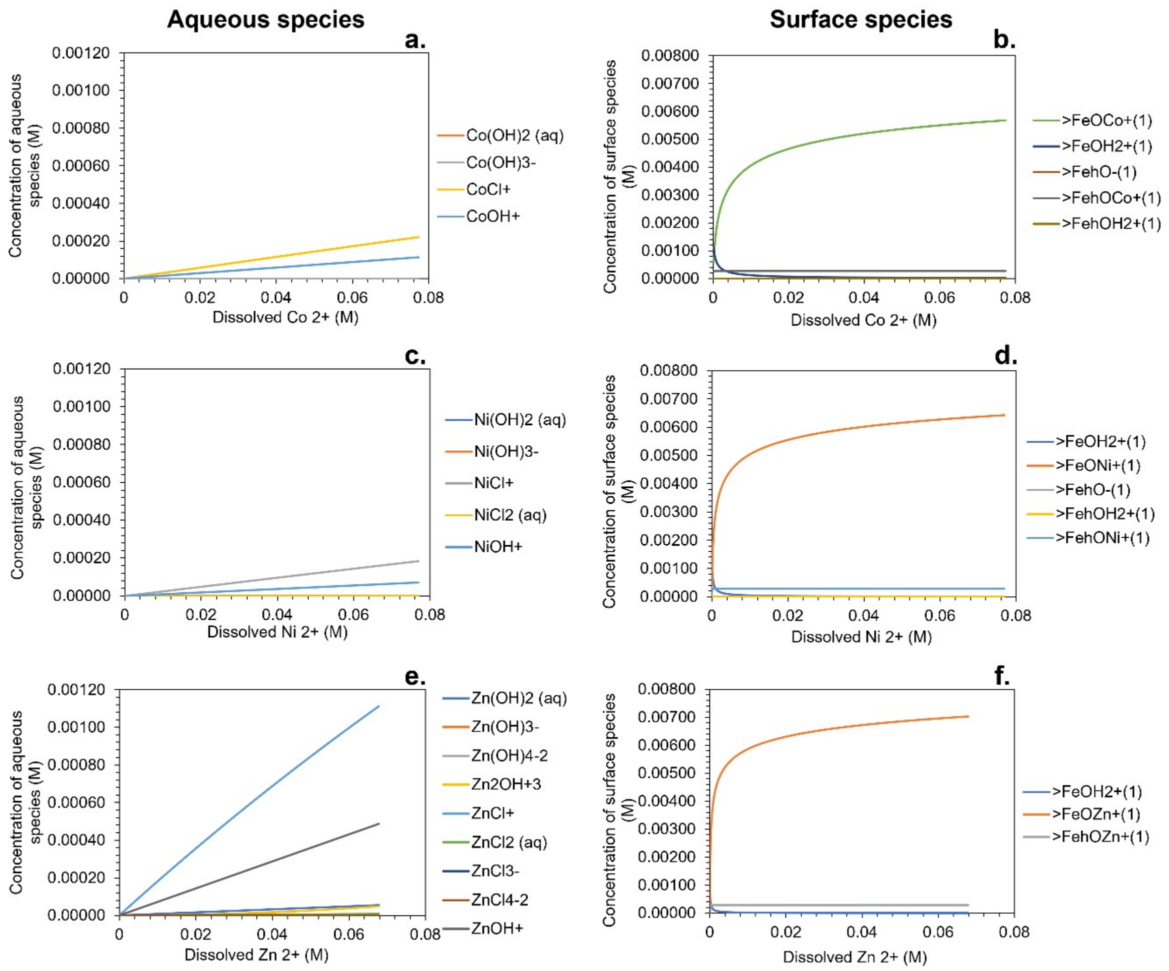

Figure S6: Sorption of Co(II), Ni(II) and Zn(II) in separate experiments on HFO model (5 g L^{-1}) at pH 7. In Figure 6a, c and concentration of aqueous species (y axis) and dissolved initial concentration (x axis) for a) Co(II), c) Ni(II) and e) Zn(II). Whereas Figure 6b, d and f displays the concentration of surface species (y axis) and dissolved initial concentration (x axis). Models were generated using Visual MINTEQ 3.1.

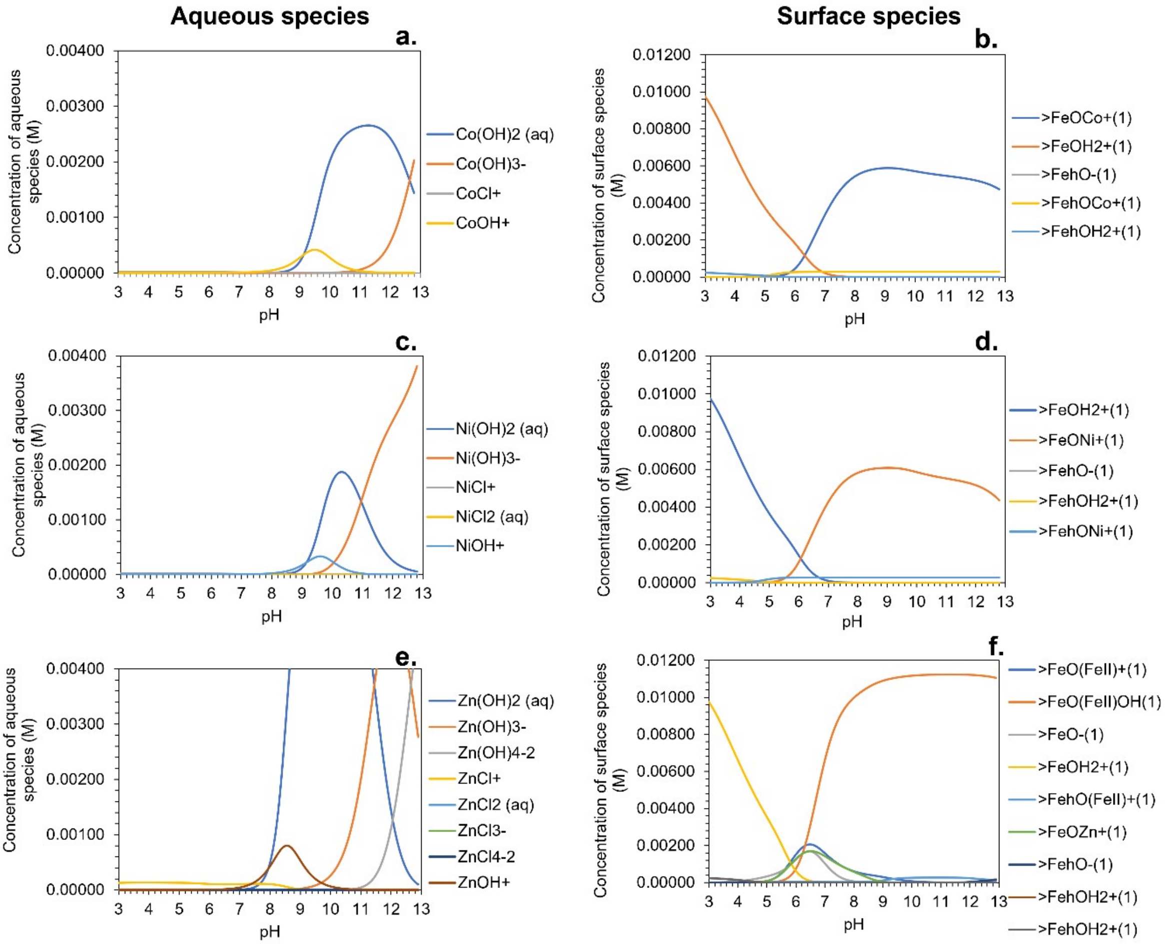

Figure S7: Sorption of Co(II), Ni(II) and Zn(II) in separate experiments on HFO model (iron 5 g L^{-1}). In Figure 7a, c and e, concentration of aqueous species (y axis) and pH ranged from 3 to 13 (x axis) for a) Co(II), c) Ni(II) and e) Zn(II). Whereas Figure 7b, d and f displays the concentration of surface species (y axis) and pH (x axis). Models were generated using Visual MINTEQ 3.1.
